# Bullying Victims in Rural Primary Schools: Prevalence, Correlates, and Consequences

**DOI:** 10.3390/ijerph19020765

**Published:** 2022-01-11

**Authors:** Huan Wang, Jingjing Tang, Sarah-Eve Dill, Jiusi Xiao, Matthew Boswell, Claire Cousineau, Scott Rozelle

**Affiliations:** 1 Stanford Center on China’s Economy and Institutions, Stanford University, 616 Serra Mall E501, Encina Hall, Stanford, CA 94305, USA; huanw@stanford.edu (H.W.); sedill@stanford.edu (S.-E.D.); kefka@stanford.edu (M.B.); clairece@stanford.edu (C.C.); rozelle@stanford.edu (S.R.); 2 Department of Economic Sciences, Computational Justice Lab, Claremont Graduate University, 150 E 10th Street, Claremont, CA 91711, USA; xiao.josie@gmail.com

**Keywords:** bullying, rural China, educational performance, creativity

## Abstract

School bullying is a widely recognized problem in developed countries, but remains under-investigated in developing countries, especially in remote rural areas. In this paper, we examine the prevalence, correlates, and consequences of bullying victimization and its relation to educational performance and creative attitudes. Using data from 10,528 students across 120 primary schools in rural China, we find an alarmingly high prevalence of bullying victimization and that several individual, family, and school characteristics are correlated with bullying victimization. Analyses indicate students who are bullied frequently score lower in Chinese, reading, and math tests and creative attitudes. Taken together, the results demonstrate a need for further research and policy interventions to reduce bullying in schools.

## 1. Introduction

While school-based violence can take many forms, bullying is particularly prevalent. Despite definitions varying, bullying is generally considered to be “intentional repeated overt or covert inappropriate behavior from another that is intended to intimidate and harm the target” [1]. In school settings, a student is bullied “when he or she is exposed, repeatedly and over time, to negative actions on the part of one or more other students” [2]. For example, negative actions, or bullying, can be direct or indirect and include physical, verbal, or psychological and relational acts that cause children to be systematically excluded from social activities by their peers [3,4].

In many countries, rates of bullying are high. According to a cross-national study of adolescents aged 11.5 to 15.5 years, the prevalence of bullying victimization (a term henceforth used to indicate that a child is a victim of bullying) ranges from 6% in Sweden to 40% in Lithuania, with an average rate of 17% across 25 countries in Europe and North America [5]. Another cross-national study, the Global School-based Student Health Survey (GSHS), carried out among middle school students in 19 low- or middle-income countries showed that the prevalence of bullying ranged from 8% in Tajikistan to 61% in Zambia [6]. In the 2011 Progress in Reading and Literacy Study (PIRLS), which draws on a sample of fourth grade students spanning 52 countries and regions, about 53% of students reported that they had been bullied at school, and 20% said that they were bullied “almost weekly” [7].

Bullying has been shown to have significant consequences on a student’s educational experience and long-term outcomes [8,9]. Studies have found that being bullied at school causes school avoidance and poor attendance [10], inability to concentrate [11,12], lack of academic engagement [13,14,15], early school dropout [16], and a weaker sense of belonging or connection with school [17]. In addition to academic measures, school environments may have an influence on student creativity [18], and bullying has been found to detrimentally affect school environments [19,20]. As such, in addition to the immediate psychological and social consequences, the negative impact of bullying on educational performance can inhibit human capital accumulation, labor market opportunities, and economic productivity in the long-term [21,22,23].

In China, little is known about school-based bullying. The available research, which draws upon samples that are almost exclusively urban, finds that the prevalence of self-reported bullying victimization varies from 2% to 26% [24,25,26,27,28,29]. Similarly, studies of the correlates of bullying in urban China have varied widely. While some studies show that bullying victims in China tend to be male students [25,27,28], a study in Tianjin finds no indicative gender pattern in bullying victimization [30]. Other evidence suggests that the gender of teachers was associated with their responses to student bullying behaviors in school [31,32,33]. In addition, some research reports that students from single-parent families had significantly higher rates of bullying victimization than children from two-parent families [34,35]. However, another study shows that students who were dissatisfied with their parental caring, not students who experienced parental absence, were at a higher risk of being bullied [27].

Although existing studies have facilitated an understanding of bullying victimization in urban China, the existing research provides little insight into the extent of the bullying problem in China’s vast rural school system, where most of the country’s children attend school. This area of inquiry is particularly important given that rural students lag far behind their urban counterparts in academic achievement and attainment [36,37,38]. To the extent that bullying negatively impacts the social emotional well-being of students, as well as their educational performance and creativity (as seen in the literature discussed above [8,9,10,11,12,13,14,15,16,17,18,19,20,21,22,23,24]), an understanding of bullying in rural schools is an important step in narrowing the rural-urban gap in China’s education system. China is currently attempting to grow from an upper middle-income economy to a high-income economy, but insufficient human capital in rural areas threatens this transition [36]. As the government is exerting considerable efforts to improve human capital in both urban and rural areas [39], understanding the various causes of rural academic underachievement is crucial to effectively narrowing the rural-urban gap. A more thorough understanding of the prevalence, correlates, and consequences of bullying victimization in rural China will not only inform efforts to reduce bullying and improve student well-being; it will also offer insights into how to improve human capital accumulation in rural China and other developing contexts.

The overall goal of this study is to examine the prevalence, correlates, and consequences of bullying victimization in China’s rural schools, focusing on its relationship to educational performance and creative attitudes. More specifically, the hypothesis we would like to test in this paper is the following: bullying victimization is correlated with lower levels of academic performance and lower levels of creative attitudes among rural students in China. To achieve this goal, we pursue three objectives. First, we document the prevalence of bullying among primary school students in rural China and compare this to other countries using an international comparative metric from the Trends in International Mathematics and Science Study (TIMSS) and Progress in Reading and Literacy Study (PIRLS) survey [40,41,42,43,44]. Second, we identify the student family and school characteristics that are correlated with bullying victimization. Third, we examine whether bullying victimization is correlated with student academic performance and student creative attitudes both before and after controlling for school, student, and family characteristics.

The rest of the paper is organized as follows. Section 2 introduces sampling methods, data collection, and methods for measuring bullying victimization, academic performance, and creative attitudes. Section 3 reports the prevalence and correlates of bullying victimization and the relationship between bullying victimization, academic performance, and creative attitudes. Section 4 discusses the results. Section 5 concludes.

## 2. Materials and Methods

### 2.1. Sampling

The data presented in this study were collected from three rural counties in the southern part of Jiangxi Province in China (henceforth referred to as Counties A, B, and C). Although our sample is from one province of China, it is fairly representative of poor rural counties across China in terms of key economic and social indicators. First, all three sample counties are nationally designated poor counties that were identified by the Chinese government in 2012 as areas with extreme poverty (among other data, the indicators used to identify poor counties include per capita GDP, per capita general budgetary revenue, and rural per capita income) [45]. As such, the economic development in these three counties lags behind the national average in China as well as other areas of Jiangxi Province. Per capita disposable income in each of the three counties was less than RMB 8200 (USD 1280) in 2015, which is similar to the average per capita disposable income of RMB 9264 (USD 1447) in the 832 nationally designated poor rural counties across China [46]. Additionally, more than 80% of the population in the three counties are rural residents [46]. In these respects, the three sample counties are typical of poor rural areas across China, which are home to nearly one fifth of China’s total population.

To select our sample, we followed a two-step sample selection protocol. The first step involved selecting a representative sample of schools from the three counties. To do so, we used official records from county education bureaus to create a population frame of all rural, public primary schools in the three counties, totaling 458 schools. We then randomly selected schools using a sampling fraction that ensured the total number of schools in each township was proportionally represented in our sample. This led us to randomly select 120 schools, of which 37 (30.9%) were in County A, 25 (20.8%) were in County B, and 58 (48.3%) were in County C (Table 1). In this way, our sample is representative of the three counties being studied.

After selecting schools, we next sampled classes and students in grades four and five. Due to financial constraints, we randomly selected at most two classes in each grade in each school. Specifically, if there were one or two classes in a grade, all classes in this grade were selected. If there were more than two classes in a grade, we randomly selected two classes. We then surveyed all students in the sampled classes. We also surveyed the math teacher and Chinese teacher for each sample class. Our final sample included 10,528 students from 286 classes in our 120 sample schools (Table 1). This sample is far larger than previous samples that have been used to examine bullying in Chinese schools [26,27,29].

### 2.2. Data Collection

All data collections were conducted at the end of the school year in May 2015. We collected four blocks of data. The first block collected information on bullying victimization in school. The second block collected socioeconomic information about students, households, and schools/teachers. The third and fourth blocks collected data on student academic performance and student creative attitudes, respectively.

#### 2.2.1. Bullying Victimization

To collect information on student bullying victimization among our sample, we used the “Students Bullied at School” (SBS) scale. The SBS scale was developed for the Trends in International Mathematics and Science Study (TIMSS) and Progress in Reading and Literacy Study (PIRLS) [40,41,42,43,44]. Both the TIMSS and PIRLS are international comparative assessments of academic achievement among fourth grade students across 52 countries and regions representing a variety of development and income levels [7]. The SBS survey was translated into Mandarin Chinese and the translation was verified according to the PIRLS translation guidelines [47]. The SBS scale also has good reliability among teachers in rural China with a Cronbach’s alpha reliability coefficient of 0.93.

The SBS scale asks students to rate how often they experienced each of six bullying victimization behaviors. The six behaviors are (a) I was made fun of or called names; (b) I was left out of games or activities by other students; (c) Someone spread lies about me; (d) Something was stolen from me; (e) I was hit or hurt by other student(s) (e.g., shoving, hitting, kicking); and (f) I was made to do things I didn’t want to do by other students. To create a raw score for the SBS scale, each response was assigned a numerical value (“at least once a week” = 0, “once or twice a week” = 1, “once or twice a month” = 2, and “never” = 3). The raw scores range from 0 (suffers all six kinds of bullying at least once a week) to 18 (never suffers any of the six kinds of bullying). A lower SBS score therefore corresponds to a higher level of bullying victimization in school. Following the PIRLS protocol, raw scores were transformed into SBS scaled scores, which were then used to sort students into three categories by frequency of bullying victimization: “Almost Never,” “About Monthly,” and “About Weekly.” Transformed SBS scale scores range from 3 to 13 points. PIRLS guidelines categorize students with transformed scores higher than 10.1 as “Almost Never” bullied; students with transformed scores between 8.3 to 10.1 as bullied “About Monthly;” and students with transformed scores below 8.3 as bullied “About Weekly.” Students experience bullying victimization behaviors “about weekly” and “about monthly” are considered to be “frequently bullied”.

#### 2.2.2. Socioeconomic Information

The survey team collected data on the basic socioeconomic information of each student, as well as information about each student’s family, teacher, and school. Student socioeconomic information included gender, grade, and a seven-item checklist of household assets. The checklist asked students to indicate whether the family owned a car, a microwave, a refrigerator, a camera, a computer, an electric fan, and/or a flush toilet. A value was attached to each asset (based on the National Household Income and Expenditure Survey, which is organized and published by the China National Bureau of Statistics—CNBS, 2008) to produce a single metric of household asset holdings. Summing the value of all household consumption assets then produced our proxy variable for family asset value. Enumerators also asked students about where they lived during most of the school year—at home or in the school’s boarding dormitories (in many rural school districts, a significant number of primary school students live in dormitories at school due to the national school merger program that shut down village schools and built a number of centralized boarding schools in towns and large villages [48,49]). In addition, a survey form was sent to each student’s caregivers to collect data on parental education levels, and on whether the student’s parents were often away from home. Finally, we surveyed teachers and principals to collect data on the gender of Chinese and math teachers in each class, the student–teacher ratio of each school, and the school’s distance from the local government seat.

#### 2.2.3. Academic Performance Tests

We conducted a set of academic performance tests, including a 30-min standardized reading test, a 30-min standardized Chinese language test, and a 30-min standardized mathematics test. All sample students were administered the reading test, which was carefully designed to measure student reading skills. The test questions were adapted from those found in the PIRLS test. The test questions were carefully translated according to the PIRLS translation guidelines and reviewed by a panel of experts and local teachers who are well-versed in China’s education system. The translated reading tests then went through several rounds of pilot tests in Chinese schools. The results were independently reviewed by a group of test assessment experts and were revised to make sure they were of the highest quality and appropriate for the designated student levels.

In addition to the reading test, students were administered mathematics and Chinese language tests. The tests evaluating math and Chinese language were carefully designed with assistance from educators in the local education bureaus to ensure coherence with the national curriculum, and both exams were pre-tested multiple times to confirm their academic relevance and appropriate time limits. Within each selected school, we randomly assigned half of the sample students in each classroom to take the math test; the other half of sample students in each classroom took the Chinese test. In total, 5237 students (49.7% of the total sample) took the reading and Chinese language tests, while 5291 students (50.3%) took the reading and math tests. Trained enumerators proctored all exams to prevent cheating and enforce the 30-min time limit. For ease of interpretation, we converted all test scores into z-scores using the mean and standard deviation of scores in each grade.

#### 2.2.4. Creative Attitudes

In the final survey block, students were asked to complete the Schaefer’s Creativity Attitude Survey [50]. This instrument includes 32 questions designed to assess children’s attitudes associated with creativity, such as confidence in one’s ideas, appreciation of fantasy, openness to impulse expression, and use of novelty [50]. Students were given unlimited time to complete the survey. The total score ranges from 0 to 20, with higher scores indicating greater creativity.

### 2.3. Analytical Approach

Our analysis is comprised of three parts. First, in our initial analysis, we looked at the prevalence of bullying at school. Second, to understand what kinds of students are more likely to be bullied, we compared the rate of bullying victimization (percent of students who are bullied at school almost weekly and almost monthly according to the six subcategories of the SBS scale) with different individual, family, teacher, and school characteristics. We conducted t-tests to measure for significant differences between groups and to analyze which characteristics correlate with bullying victim status.

Finally, we estimated the correlation between bullying victim status and academic performance, as well as the correlation between bullying victim status and creative attitudes. To do so, we used an ordinary least squares (OLS) regression model, including a set of covariates in a regression on student dropout. We first ran an unadjusted regression (1):

(1) $$Y_{ij}=\alpha +\beta Bullied_{i}+\varphi_{j}+\varepsilon_{i}$$

where the dependent variable $Y_{ij}$ denotes the academic performance (including standardized scores in reading, Chinese, and mathematics) and creative attitudes of student j in school k. $Bullied_{i}$ is a dichotomous variable that equals 1 if the student is being bullied at school about weekly or about monthly, and equals 0 if the student is almost never bullied at school; $\varphi_{j}$ represents county-level fixed effects; and $\varepsilon_{i}$ is an error term capturing shocks and characteristics that are specific to the student or are unobserved. β is the within-school mean gap in academic performance and creative attitudes between bullying victims and non-bullied students.

To control for the potential confounding effects of student, family, teacher, and school characteristics, we ran a multivariate analysis building on Equation (1) above with the addition of a vector of control variables.

(2) $$Y_{ij}=\alpha +\beta Bullied_{i}+\gamma X_{i}+\varphi_{j}+\varepsilon_{i}\;$$

where the vector $X_{i}$ includes student, family and teacher, and school characteristics. Student individual characteristics include binary variables representing student gender, grade, and boarding status. Family characteristics include: household wealth (variable equals 1 for households in the lowest quartile and 0 for households in the top three quartiles), type of family (1 if the student is from a single- or zero-parent family, 0 if the student is from a two-parent family), whether parents graduated from junior high school, and whether parents are often away from home. We control for Chinese teacher gender when the outcome variable is Chinese or reading performance, for the mathematics teacher gender when the outcome variable is mathematics performance, and for the gender of both teachers when the outcome variable is creative attitudes. School characteristics include the student–teacher ratio (variable equals 1 if the student–teacher ratio is in the lowest quartile with fewer teachers per student, 0 if the student–teacher ratio is in the top three quartiles), and distance between the school and government seat (1 if the distance is in the farthest quartile, 0 if in the nearest three quartiles).

We chose to include the aforementioned variables in our equation based on previous studies that identified them as important factors correlated with student academic achievement. Past research has indicated that individual [51], family [48,51], teacher, and school characteristics [52] are all closely related to the academic performance of students. Therefore, controlling for these variables allows us to better isolate the effects of bullying on student achievement and compare the magnitude with other factors. In both Equations (1) and (2), we computed cluster-robust standard errors (adjusted for clustering at the school level).

## 3. Results

### 3.1. Prevalence of Bullying among Primary School Students in Rural China

**Figure 1:**
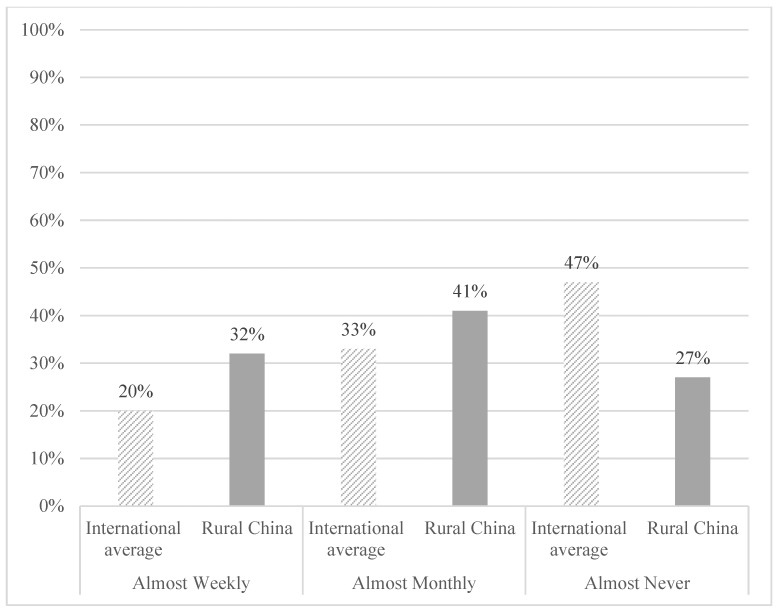
Comparison of prevalence of bullying victimization between rural China and international average proportion.

Figure 1 presents the rates of bullying victimization among students in our sample. Our results show that the prevalence of bullying victimization among primary school students in rural China is alarmingly high. Seventy three percent (73%) of sample students were bullied frequently (defined as either monthly or weekly). This is a significantly higher rate of bullying than the average rate across 52 countries reported by the TIMSS and PIRLS survey (using an identical scale as our study), in which 53% of students experienced bullying weekly or monthly [3]. The prevalence of bullying in our sample is also substantially higher than previous estimates of bullying in China. Previous studies have found that the prevalence of bullying is around 20% in Hong Kong [53,54] and 2% to 26% in urban areas of mainland China [24,25,26,27,28,29], meaning that the prevalence of bullying in our sample is nearly three times higher than the highest prevalence reported in studies of urban China. In addition, among our sample, 41% of student reported being bullied almost monthly, and 32% reported being bullied almost weekly. This is also much higher than the rates reported in the 2011 TIMSS and PIRLS survey, which found that only 33% of students experienced bullying monthly and only 20% were bullied weekly [7].

**Figure 2:**
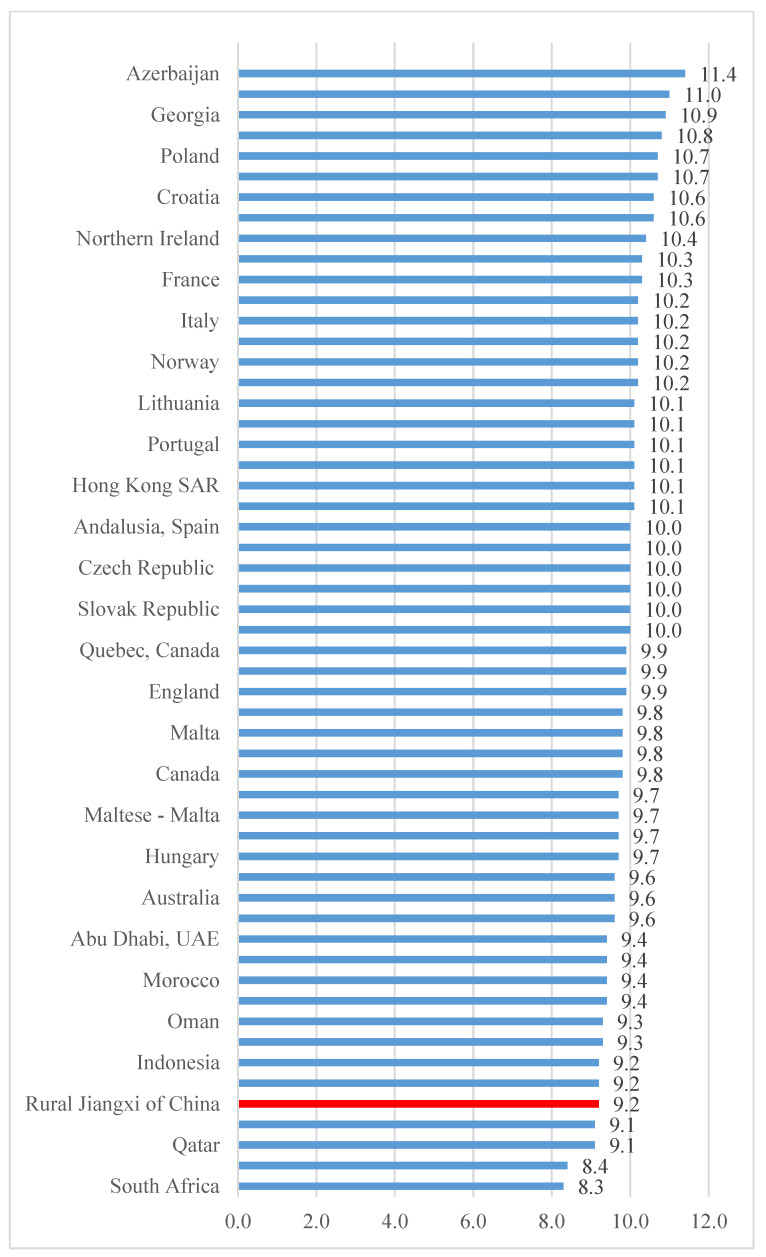
Comparison of average students being bullied at school (SBS) scale scores between rural China and other countries/regions. Note: a lower SBS score corresponds with a higher level of bullying victimization in school. To enable comparison across countries and regions, SBS raw scores have been converted to scales scores according to the PIRLS conversion chart.

In comparison to the bullying victimization scale score of other countries and regions, our results show that students in rural China experience bullying more frequently than students in most other places in the world. The average SBS scale score (where a higher score means less bullying) for our sample of rural students is 9.2, meaning rural Chinese students are categorized as “being bullied monthly”, according to the TIMSS and PIRLS survey cutoff. This puts our sample region at a rank of 5th out of 52 countries or regions, with only slightly lower rates of bullying than South Africa, Botswana, Qatar, and Trinidad and Tobago (Figure 2).

When we examine each of the six bullying behaviors assessed in the SBS scale, we find that certain types of bullying were reported more frequently than others among our sample students. The most common types of bullying that students experienced were “being made fun of or called names”, “being stolen from”, and “exclusion from games or activities by other students” (Table 2). Of our sample students, 73% had been made fun of or called names at least a few times in the past year; 73% had had personal items stolen from them at least a few times in the past year; and 58% had been excluded by other students at least a few times in the past year. The three other bullying behaviors included in the SBS scale were also fairly commonplace among sample students: in the past year, 50% of students had experienced lies being spread about them at least a few times; 50% had been hit or hurt by other students at least a few times; and 41% had been forced to do things they did not want to do at least a few times.

### 3.2. Factors Correlated with Bullying Victimization

Table 3 presents the results of our analysis of the characteristics associated with bullying victimization. We found that several student characteristics were associated with higher rates of bullying victimization, namely gender, age, and boarding status. Boys were slightly more likely to be bullied than girls (74% compared to 71%, significant at the 1% level—Table 3, row 1). In addition, grade five students reported being bullied at a slightly higher rate than grade four students (74% compared to 72%, significant at the 5% level—Table 3, row 2). Students who board at school also experienced significantly more bullying in comparison to students who live at home (83% compared to 72%, significant at the 1% level—Table 3, row 3). However, it is important to note that although these differences are statistically significant, the rates of bullying victimization for all groups are far higher than the international average.

Regarding household and school characteristics, several variables associated with socioeconomic disadvantage were significantly correlated with more frequent experiences of being bullied. Students in the lowest quartile of family asset value, students from single parent families, and students whose fathers or mothers did not finish junior high school were more likely to experience bullying victimization (significant at the 1% level—Table 3, rows 4–7). Interestingly, there was no significant difference in bullying victimization between the left behind children and students whose parents lived at home (Table 3, rows 8 and 9). We also found that remote schools were associated with higher bullying rates, and students at schools with higher student–teacher ratios experienced more bullying than students at schools with lower student–teacher ratios (74% compared to 69%, significant at the 1% level—Table 3, row 12). However, in contrast with previous studies [31,32,33], our results find no association between the gender of teachers and students bullying victimization.

To further investigate the correlates of bullying victimization, we ran a multivariate regression that includes all student, family, teacher, and school characteristics (Table 4). The results are overall consistent with that of Table 3. Of the individual student characteristics, boys and boarding students tended to be bullied more frequently (Table 4, rows 1 and 3). Our finding that boarding students experienced more frequent bullying is consistent with past findings in urban China and in other countries that boarding students show higher levels of bullying victimization and perpetration in comparison to non-boarders [55,56,57]. As Pfeiffer and Pinquart discuss, this may be because students who board at school spend more time with peers, for example, in dormitories [55]. Similarly, Chui and Chan suggest that more peer contact, especially with deviant peers in dormitories, contributes to bullying victimization [57]. This is certainly the case in rural Chinese boarding schools, where students may share a dormitory room with more than ten peers [58]. Moreover, separation from parental influence may reduce the impact of parenting practices meant to reduce problem behavior, leading students to engage in more bullying behaviors [55].

Additionally, the results of our multivariate regression show that students whose families have the lowest family asset values, students in single-parent households, and student whose mothers or fathers have not completed junior high school were more likely to experience frequent bullying (Table 4, rows 4–7). These findings are consistent with previous studies that have found children and adolescents from families of lower socioeconomic status are more likely to be involved in bullying victimization [59,60,61,62]. Low family wealth itself may be one cause of victimization, as students have less access to financial resources and inability to afford material goods [35,62]. Additionally, as Jansen et al. [63] suggest, students from single-parent households are more likely to experience an increased level of stress due to broken families and fewer parent-child interactions. The latter explanation is further supported by the evidence presented in Spriggs et al. that found a reduction in parental involvement and communication to be associated with increased bullying victimization [64]. Among our sample, the higher magnitude and significance of single-parenthood than family asset value implies that parenting characteristics associated with single parent households are contributing factors to the risks of bullying victimization.

The literature also supports our finding that students whose parents have lower education levels are at greater risk of being frequently bullied. Research has shown that there is an inverse relationship between parental education levels and child bullying victimization, especially maternal education levels [60,65,66,67]. Together with family asset value and single-parenthood, parental education levels serve as a socioeconomic status indicator [63]. In addition to financial resources, parents of higher socioeconomic status can provide more time, knowledge, and aid to help their children cope with social conflicts. Both aspects reduce the chances that children will be bullied. This point is further supported by the statistically insignificant relationship between whether the father or mother lives at home and bullying victimization. The mere presence of parents at home, when other family factors are controlled for, does not have a significant impact on whether a child is bullied. Instead, it is the underlying socioeconomic status of the family, determined in large part by parental education, which matters.

Finally, we found that students at schools with higher student–teacher ratios tended to be bullied more frequently (a difference of 3.5 percentage points, significant at the 10% level; Table 4, row 12). Frequent bullying victimization has been found in the literature to be associated with a large school size [68,69] and a high student–teacher ratio [70]. This may be because high student–teacher ratios limit teachers from effectively managing student behavior and preventing bullying victimization [70].

### 3.3. Correlations between Bullying Victimization, Academic Performance, and Creative Attitudes

Table 5 presents the results of our OLS regression analysis examining the correlation between bullying victimization and academic performance. Our unadjusted OLS regression results (columns 1, 2, and 3) show that experiencing frequent bullying is significantly correlated with lower academic performance. Being bullied monthly or weekly is associated with a decrease of about 0.26 standardized deviations in Chinese language performance, 0.25 standardized deviations in reading performance, and 0.22 standardized deviations in mathematics performance. Our adjusted OLS regression results (columns 4, 5, and 6) tell a similar story: frequent bullying victimization is associated with a decrease of about 0.21 standardized deviations in Chinese language performance, 0.22 standardized deviations in reading performance, and 0.21 standardized deviations in mathematics performance. The magnitude of the effect of bullying victim on student academic performance is equivalent to almost half a year of learning [71].

Additionally, we find a significant negative correlation between bullying victimization and student creative attitudes (Table 6). Students who were bullied weekly or monthly scored lower in creative attitudes than their peers in both the unadjusted and adjusted models, with coefficients of 0.60 and 0.57, respectively (Table 6, columns 1 and 2). This corresponds to a difference in creative attitudes of about 0.24 standard deviations. The findings in both Table 5 and Table 6 are all significant at the 1% level. In other words, the magnitude of these associations and their statistical significance are similar in the non-adjusted and adjusted regressions for both academic performance and creative attitudes.

To better understand the relative “importance” of bullying for academic performance and creativity, we compare the magnitude of bullying victimization to other significant factors in our multivariate analysis. Of note, the magnitude of the correlation between bullying victimization and student academic performance is as large as the gender gap and single-parenthood, and it is greater than that of boarding status, low parental education and absent fathers. Moreover, the magnitude of bullying victimization on student creativity ability is greater than all other factors measured in this study, including gender, boarding status, low family asset value, single-parenthood, low parental education levels, remote school locations and high student–teacher ratios.

These results confirm our initial hypothesis that bullying victimization has a negative impact on academic performance, consistent with the findings of previous studies [8]. More specifically, the literature has found that when a student experiences bullying, the stress incurred from victimization can lead to school avoidance, poor class attendance, and the inability to concentrate in class, all of which directly impede learning and academic achievement [10,11,12]. Similarly, the creative attitudes of students are hampered when they experience bullying. As previous research suggests, positive and encouraging environments are more likely to foster creativity [72,73]. The stress and reduced self-esteem incurred from bullying victimization might create a hostile environment that inhibits student creativity. Moreover, when we compare the magnitudes of correlation, we find that bullying victimization is negatively correlated with academic performance and creative attitudes at a larger magnitude than many other factors. This suggests that reducing bullying in rural schools may have a larger positive effect on student academic performance and creative attitudes than targeting other factors that have been traditionally considered.

## 4. Discussion

To the best of our knowledge, ours is the first large-scale study to document the prevalence of bullying victimization among students in rural China. This study also identifies the student individual, family, and school characteristics correlated with bullying victimization. Additionally, this is the first study to examine the correlation between bullying victimization and student educational performance in rural China, before and after controlling for student and family characteristics.

Our study finds that students are bullied in rural Chinese elementary schools at rates far higher than both the international average and the rates found in urban areas of China. About 73% of sample students in rural China were bullied almost monthly or weekly, which is much higher than the international average of only 53%. The prevalence of bullying in rural China is also much higher than that of Hong Kong and urban areas of mainland China, where rates of bullying vary from 2% to 26% [24,25,26,27,28,29,53,54]. The most frequently experienced type of bullying in rural China is being made fun of or called names by other students. We also found several characteristics predictive of frequent bullying victimization, including being male, boarding at school, having less educated parents, having a family with a lower family asset value, having a single-parent family, and attending a school with a higher student–teacher ratio.

Perhaps most importantly, even after controlling for student, family, teacher, and school characteristics, student academic performance and creative attitudes are both strongly negatively correlated with frequent experiences of bullying. A student being bullied monthly or weekly is correlated with a decrease of about 0.21 standardized deviations in Chinese performance, 0.22 standardized deviations in reading performance, 0.21 standardized deviations in mathematics performance, and 0.57 points (0.24 standardized deviations) in creative attitudes. In other words, bullying significantly impedes the ability of students to perform academically and think creatively.

Why do we see such high rates of bullying victimization in rural China? One factor may be that rural teachers lack the time and resources to intervene and prevent bullying. With China’s rapid economic progress and intensified urbanization, more financial and educational resources have been poured into cities. Rural areas, though on the radar of policymakers, still face a scarcity of educational resources [74,75]. Additionally, fiscal decentralization has aggravated the unequal distribution of resources in rural areas, leading to low per-pupil basic education expenditures in rural schools [76,77,78]. This means that rural teachers face lower salaries, worse working conditions, heavier workloads, and limited school budgets for professional development compared to their urban colleagues, all of which lead teachers to move within the public education system to better-paying urban schools, creating a shortage of rural teachers [79,80]. As a result, rural schools tend to have larger class sizes and higher student–teacher ratios, leading to less individualized attention for each student. This can explain in part the observed high prevalence of bullying victimization. Especially in an exam-centric education system such as in China, where the exam scores are the primary metrics for academic performance, teachers tend to focus their limited time and effort on teaching and student academic achievement rather than behavior management.

Another key factor may be parenting quality and socioeconomic challenges in rural China. Low socioeconomic power in rural families creates adversities for rural children. For example, many rural parents leave rural areas for jobs in China’s cities and leave their children behind in the countryside in the care of grandparents. The absence of parents and reduced parental involvement deprives children of opportunities to learn conflict management skills and other related social skills [61]. Moreover, grandparents often have even lower levels of education, which means that can only provide limited guidance to address the troubles in their children’s social lives [81,82,83].

Bearing the disadvantages of both educational resources and parental attention, rural students face a higher level of bullying victimization. The negative consequences such as stress hinder not only their academic performance but also limit their creative abilities. This, in turn, perpetuates the preexisting urban-rural educational divide.

## 5. Conclusions

The findings in this paper offer insights into factors that may contribute to student academic performance and creative attitudes in rural China beyond those that have been traditionally considered, such as teacher quality, school funding, and student physical health. While our data do not support a causal analysis of the relationships among bullying, academic performance, and creative attitudes in rural China, studies conducted in other countries can provide guidance for future causal research in China. Specifically, many studies find that bullying has negative consequences for a student’s educational experience and long-term outcomes [8,9,10,11,12,13,14,15,16,17,18,19,20,21,22,23], disrupting their ability to learn, think, and thrive in the school environment. Future research into the effects of bullying in rural China should therefore focus on the causal links between bullying and physical/mental health.

The results of this study also have implications for China outside of its academic system. If bullying victimization does in fact has a significant negative impact on student academic performance and creative attitudes, then widespread bullying may not only be hurting individual students; it may also harm China’s long-term economic development by slowing human capital growth. Supposing that the proportion of students found to be frequently bullied (73%) holds true across rural China, then based on the Ministry of Education’s statistic that there are about 30 million primary school students in rural China, 22 million primary school students are bullied frequently in China, at the expense of their academic achievement. Given the important link between education and human capital, the correlation between bullying and academic performance offers a direction for improving human capital in rural areas, where widespread low academic performance and high rates of dropout negatively affect educational attainment and labor market performance among large parts of the population.

Given these findings, we recommend that China’s education policymakers consider incorporating bullying research and prevention initiatives into their agenda. Bullying is a complex and pervasive phenomenon, and it is necessary to understand the problem in full, especially in poor rural areas where academic performance lags in comparison to urban areas. Therefore, causal studies of bullying and its effects on students are vitally important. In addition, although self-reported data on bullying victimization may reveal the true prevalence and magnitude of bullying behavior, future research should explore the characteristics of rural bullies using direct observation methods, as understanding the profile of bullies is critical in addressing bullying more broadly. Anti-bullying programs should also be piloted throughout rural schools. Furthermore, funding for controlled trials of such programs should be prioritized so that there is a solid evidence base for developing anti-bullying policies in the future.

## Figures and Tables

**Table 1:** Sample distribution.

County	Number of Schools	Percentage	Number of Chinese Teachers	Percentage	Number of Math Teachers	Percentage	Number of Students	Percentage
County A	37	30.9	97	33.9	97	33.9	3962	37.6
County B	25	20.8	55	19.2	55	19.2	1603	15.2
County C	58	48.3	134	46.9	134	46.9	4963	47.2
Total	120		286		286		10528	

Data source: Authors’ survey.

**Table 2:** Prevalence of bullying victimization in rural China (by items, percentage).

Student Being Bullied at School Scale	At Least Once a Week	Once or Twice a Month	A Few Times a Year	Never
(1) I was made fun of or called names	32.89	17.91	22.21	26.99
(2) I was left out of games or activities by other students	18.67	16.42	22.60	42.31
(3) Someone spread lies about me	15.57	14.03	20.74	49.66
(4) Something was stolen from me	16.15	20.83	35.61	27.41
(5) I was hit or hurt by other student(s) (e.g., shoving, hitting, kicking)	13.82	13.62	22.42	50.14
(6) I was made to do things I didn’t want to do by other students	10.79	11.04	19.62	58.55

Note: the six statements were developed by the Progress in International Reading Literacy Study (PIRLS) in 2011 and they are used to measure students being bullied at school.

**Table 3:** Who is more likely to be bullied (monthly or weekly)?

Characteristics	Being Bullied			
	%	*N*	Difference	|t|
(1) Gender				
Male	74	5408	0.03	3.06 ***
Female	71	5120		
(2) Grade				
4th	72	5009	−0.02	2.38 **
5th	74	5519		
(3) Boarding				
Does not board	72	9549	−0.01	7.72 ***
Boards	83	979		
(4) Asset value				
In highest 25 percent	71	2450	−0.04	3.33 ***
In lowest 25 percent	75	2656		
(5) Single/zero parent family				
Both parents family	72	9128	−0.04	3.20 ***
Single/zero parent family	76	1400		
(6) Father’s education				
Not completed Junior HS	75	4562	0.04	4.07 ***
Completed Junior HS and above	71	5966		
(7) Mother’s education				
Not completed Junior HS	74	6954	0.04	4.55 ***
Completed Junior HS and above	70	3574		
(8) Whether father at home				
Usually at home	73	4332	0.01	1.51
Usually not at home	72	6196		
(9) Whether mother at home				
Usually at home	73	5195	0.01	1.07
Usually not at home	72	5333		
(10) Gender of Chinese teacher				
Male	73	4455	0.01	1.32
Female	72	6073		
(11) Gender of math teacher				
Male	73	6552	0.01	1.64
Female	72	3976		
(12) Student–teacher ratio				
In highest 25 percent	74	2623	0.05	4.02 ***
In lowest 25 percent	69	2651		
(13) Distance from local government seat				
In closest 25 percent	72	2798	−0.03	2.19 **
In farthest 25 percent	74	2549		

Note: Robust standard errors in parentheses, *** *p* < 0.01, ** *p* < 0.05.

**Table 4:** Multivariate analysis of correlates of bullying victimization.

Variables	Being Bullied (Monthly or Weekly)
	(1)
(1) Female (1 = yes)	−0.025 ***
	(0.009)
(2) Grade 5 (1 = yes)	0.013
	(0.014)
(3) Boarding (1 = yes)	0.090 ***
	(0.019)
(4) Asset in lowest 25 percent (1 = yes)	0.020 *
	(0.011)
(5) Single parent family (1 = yes)	0.037 ***
	(0.012)
(6) Father completed junior high school (1 = yes)	−0.017 *
	(0.009)
(7) Mother completed junior high school (1 = yes)	−0.025 **
	(0.010)
(8) Father usually not at home (1 = yes)	−0.011
	(0.010)
(9) Mother usually not at home (1 = yes)	−0.006
	(0.011)
(10) Chinese teacher is female (1 = yes)	−0.015
	(0.016)
(11) Math teacher is female (1 = yes)	−0.001
	(0.016)
(12) Student–teacher ratio in highest 25 percent (1 = yes)	−0.035 *
	(0.020)
(13) Remote schools in farthest 25 percent (1 = yes)	0.027
	(0.018)
County Fixed effects	Yes
Constant	0.742 ***
	(0.026)
Observations	10528
R-squared	0.017

Note: robust standard errors in parentheses *** *p* < 0.01, ** *p* < 0.05, * *p* < 0.1. Significance tests adjusted for clustering within schools.

**Table 5:** Multivariate analysis of the relationship between being bullied (monthly or weekly) and academic performance.

Variables	Chinese Score	Reading Score	Math Score	Chinese Score	Reading Score	Math Score
	(1)	(2)	(3)	(4)	(5)	(6)
(1) Being bullied (monthly or weekly)	−0.255 ***	−0.253 ***	−0.215 ***	−0.210 ***	−0.222 ***	−0.208 ***
	(0.031)	(0.028)	(0.036)	(0.030)	(0.027)	(0.035)
(2) Female (1 = yes)				0.238 ***	0.079 ***	−0.263 ***
				(0.029)	(0.020)	(0.032)
(3) Grade 5 (1 = yes)				0.010	0.008	0.010
				(0.039)	(0.028)	(0.038)
(4) Boarding(1 = yes)				−0.166 ***	−0.069	0.021
				(0.053)	(0.044)	(0.062)
(5) Asset in lowest 25 percent (1 = yes)				−0.028	−0.076 ***	−0.024
				(0.029)	(0.024)	(0.034)
(6) Single parent family (1 = yes)				−0.335 ***	−0.288 ***	−0.207 ***
				(0.041)	(0.034)	(0.039)
(7) Father completed junior high school (1 = yes)				0.223 ***	0.186 ***	0.192 ***
				(0.028)	(0.021)	(0.030)
(8) Mother completed junior high school (1 = yes)				−0.043	−0.017	0.000
				(0.030)	(0.024)	(0.030)
(9) Father usually not at home (1 = yes)				0.056 *	0.086 ***	0.098 ***
				(0.031)	(0.024)	(0.033)
(10) Mother usually not at home (1 = yes)				−0.000	0.001	0.051 *
				(0.032)	(0.023)	(0.027)
(11) Chinese teacher is female (1 = yes)				0.215 ***	0.137 ***	
				(0.049)	(0.039)	
(12) Math teacher is female (1 = yes)						0.087 *
						(0.048)
(13) Teacher-student ratio in lowest 25 percent (1 = yes)				0.062 (0.061)	0.075 * (0.043)	0.115 (0.073)
(14) Remote schools in farthest 25 percent (1 = yes)				−0.148 ** (0.061)	−0.127 *** (0.048)	−0.122 ** (0.055)
County Fixed effects	Yes	Yes	Yes	Yes	Yes	Yes
Constant	0.403 ***	0.341 ***	0.296 ***	0.068	0.131 *	0.217 **
	(0.083)	(0.066)	(0.073)	(0.101)	(0.074)	(0.085)
Observations	5237	10528	5291	5237	10528	5291
R-squared	0.056	0.036	0.032	0.116	0.070	0.077

Note: robust standard errors in parentheses *** *p* < 0.01, ** *p* < 0.05, * *p* < 0.1.

**Table 6:** Multivariate analysis of relationship between being bullied (monthly or weekly) and creative ability.

Variables	Creative Ability	
	(1)	(2)
(1) Being bullied (monthly or weekly)	−0.602 ***	−0.567 ***
	(0.067)	(0.063)
(2) Female (1 = yes)		−0.086 *
		(0.046)
(3) Grade 5 (1 = yes)		0.554 ***
		(0.092)
(4) Boarding(1 = yes)		−0.152
		(0.104)
(5) Asset in lowest 25 percent (1 = yes)		−0.217 ***
		(0.056)
(6) Single/zero parent family (1 = yes)		−0.179 ***
		(0.063)
(7) Father completed junior high school (1 = yes)		0.237 ***
		(0.045)
(8) Mother completed junior high school (1 = yes)		0.060
		(0.052)
(9) Father usually not at home (1 = yes)		0.049
		(0.062)
(10) Mother usually not at home (1 = yes)		0.008
		(0.072)
(11) Chinese teacher is female (1 = yes)		0.243 **
		(0.096)
(12) Math teacher is female (1 = yes)		0.172 *
		(0.102)
(13) Student–teacher ratio in highest 25 percent (1 = yes)		0.340 ***
		(0.113)
(14) Remote schools in farthest 25 percent (1 = yes)		−0.197 *
		(0.103)
County Fixed effects	Yes	Yes
Constant	11.680 ***	11.078 ***
	(0.119)	(0.165)
Observations	10528	10528
R-squared	0.020	0.045

Note: robust standard errors in parentheses *** *p* < 0.01, ** *p* < 0.05, * *p* < 0.1.

## Data Availability

Data are available upon request.
